# The Antimicrobial Properties of Pd^II^− and Ru^II^−pyta Complexes**

**DOI:** 10.1002/cbic.202300247

**Published:** 2023-09-05

**Authors:** Annick van Niekerk, M. Cassiem Joseph, Angela Kavanagh, Hue Dinh, Andrew J. Swarts, Selwyn F. Mapolie, Johannes Zuegg, Amy K. Cain, Alysha G. Elliott, Mark A. T. Blaskovich, Angelo Frei

**Affiliations:** ^1^ Department of Chemistry and Polymer Science University of Stellenbosch Stellenbosch, Private bag X1, Matieland 7602 South Africa; ^2^ Molecular Science Institute, School of Chemistry University of the Witwatersrand Johannesburg, PO Wits 2050 South Africa; ^3^ Centre for Superbug Solutions Institute for Molecular Bioscience The University of Queensland St. Lucia Queensland 4072 Australia; ^4^ School of Natural Sciences ARC Centre of Excellence in Synthetic Biology Macquarie University Sydney NSW 2109 Australia; ^5^ Dept. of Chemistry, Biochemistry & Pharmaceutical Sciences University of Bern Freiestrasse 3 3012 Bern Switzerland

**Keywords:** AMR, antifungal drugs, inorganic medicinal chemistry, metalloantibiotics, metals in medicine

## Abstract

Infections associated with antimicrobial resistance (AMR) are poised to become the leading cause of death in the next few decades, a scenario that can be ascribed to two phenomena: antibiotic over‐prescription and a lack of antibiotic drug development. The crowd‐sourced initiative Community for Open Antimicrobial Drug Discovery (CO‐ADD) has been testing research compounds contributed by researchers around the world to find new antimicrobials to combat AMR, and during this campaign has found that metallodrugs might be a promising, yet untapped source. To this end, we submitted 18 Pd^II^− and Ru^II^−pyridyl−1,2,3‐triazolyl complexes that were developed as catalysts to assess their antimicrobial properties. It was found that the Pd complexes, especially **Pd1**, possessed potent antifungal activity with MICs between 0.06 and 0.125 μg mL^−1^ against *Candida glabrata*. The in‐vitro studies were extended to in‐vivo studies in *Galleria mellonella* larvae, where it was established that the compounds were nontoxic. Here, we effectively demonstrate the potential of Pd^II^−pyta complexes as antifungal agents.

## Graphical abstract

**New metalloantibiotics**: With the rise and spread of multidrug‐resistant fungal pathogens, new drugs are urgently needed. In this work, new ruthenium‐ and palladium‐based compounds with promising antifungal properties are reported.

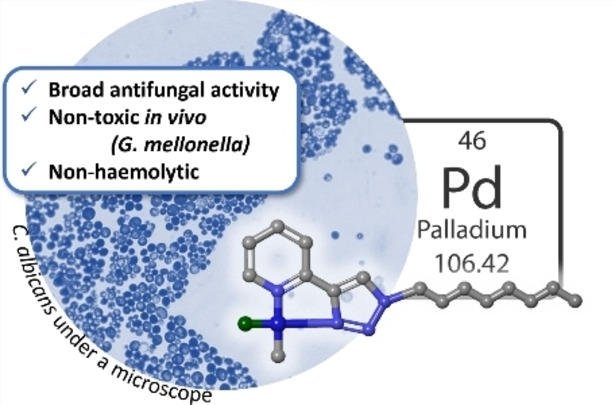

## Introduction

Antimicrobial resistance (AMR) has lived in the background of public health for many years; however, resistant infections are now on track to become the leading cause of death within the next few decades. 2019 saw an estimated 4.95 million AMR‐associated deaths,^[1]^ and this number is expected to rise rapidly in the coming years fuelled also by the indiscriminate over‐prescription of antibiotics during the COVID‐19 pandemic.^[2]^ Furthermore, treatment of COVID‐19 and HIV/AIDS often leave patients immunocompromised and therefore highly susceptible to fungal infections.^[3]^ This is of particular concern in Sub‐Saharan Africa where cryptococcal meningitis accounted for 19 % of AIDS‐related deaths in 2020.^[4]^ While most infections can be managed with current antifungal drugs —polyenes, flucytosine, azoles and echinocandins—resistance is increasing and the development of new antifungal therapies has stalled. In 2022, the World Health Organization (WHO) published the first fungal priority pathogens list, listing 19 human fungal pathogens associated with mortality or morbidity.^[5]^ In the case of disseminated cryptococcosis, no new therapies have been identified in more than 25 years,^[3]^ and with the increasing incidence of infections resistant to the current treatments, the need for new classes of therapeutics, with alternative modes of action, is greater than ever. *Candida auris* is also of significant concern, deemed of “critical importance” by the WHO report, and an “urgent threat” by the 2019 Center for Disease Control (CDC) report on AMR in the USA.

To this end, metallodrugs have shown great promise as a new class of antimicrobial agents, as outlined by Frei et al.[^6^, ^7^] in two recent publications. The crowd‐sourced organization, Community for Open Antimicrobial Drug Discovery (CO‐ADD)^[8]^ has tested over 300 000 molecules and shown that 27 % of all metal compounds screened by them showed some antimicrobial activity, compared to 2 % of organic compounds.^[9]^ Furthermore, it is likely that antimicrobial metal complexes will act through mechanisms that are distinct to those of traditional antimicrobial agents which could delay the development of resistance.

**Figure 1:**
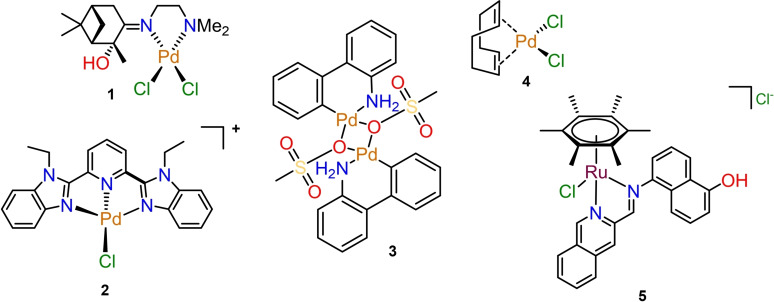
The Pd compounds (**1**–**3**) from the CO‐ADD database that displayed the highest levels of activity.^[12]^ Complex **4** was investigated by Zalevskaya et al.^[12]^ The Ru–arene complex (**5**) investigated by Weng et al.^[13]^ also displayed potent activity against MRSA.

Palladium complexes have only been explored sparsely for their antimicrobial properties.^[10]^ A recent analysis of the antifungal properties of the CO‐ADD metal complexes revealed that 51 palladium‐containing complexes were submitted for screening. Of these, 31 (61 %) showed activity against the two fungal pathogens tested by CO‐ADD, *Candida albicans* and *Cryptococcus neoformans*. Together with silver, palladium was the element with the highest number of active compounds. In addition to antimicrobial activity, CO‐ADD also tests active compounds for their cytotoxicity against mammalian cells as well as their haemolytic properties. Of the 31 active compounds, 12 palladium complexes did not show any toxic properties in these in vitro assays. Figure 1 includes three complexes (**2**–**4**) that were screened through the CO‐ADD initiative which displayed the best antifungal activity in the CO‐ADD panel.^[12]^ Zalevskaya et al. described a series of terpene‐derived palladium complexes with antimicrobial properties in 2020 of which the most promising complex, **1**, is shown in Figure 1.^[11]^ These compounds showed high levels of activity across a panel of fungal pathogens with different degrees of drug resistance. However, upon further investigation, relatively high levels of cytotoxicity and haemolysis were found for compounds **2–4**. Due to their potent antimicrobial activity, their therapeutic indices (ratio of best MIC with “worst” between CC_50_ or HC_10_) still reached as high as 2900 (for compound **2**), but due to other compounds in the library performing better, these palladium complexes were excluded from further studies.

On the other hand, ruthenium complexes have been widely investigated for their antimicrobial activities.[^7^, ^9^, ^10^, ^14^, ^15^] Notably, Weng et al.^[13]^ screened a large selection of Ru−arene Schiff‐base (RAS) complexes for antibacterial activity, where they identified complex **5** as a lead compound for further investigations. Mechanistic studies revealed that **5** was able to exert potent antibacterial activity against methicillin‐resistant *Staphylococcus aureus* (MRSA) through in situ reactive‐oxygen species induction, thereby demonstrating the potential of Ru−arene complexes as antimicrobial agents.^[13]^

As part of the collaborative effort, we submitted a selection of neutral and cationic palladium(II) pyridyl−1,2,3‐triazole complexes and cationic ruthenium(II)−pyridyl−1,2,3‐triazole complexes for antimicrobial screening. These complexes were originally synthesized and evaluated as catalyst precursors for selective ethylene dimerization^[16]^ and transfer hydrogenation[^17^, ^18^] transformations. However, they possess 2‐pyridyl−1,2,3‐triazole (pyta) moieties which have shown biological activity on their own^[19]^ and when coordinated to various metal centres in previous studies, particularly to Re^I^ for applications as theranostics.[^20^, ^21^, ^22^, ^23^]

Here we report the in vitro screening of these complexes against the ESKAPE pathogens (*Enterococcus faecium*, *S. aureus*, *Klebsiella pneumoniae*, *Acinetobacter baumannii*, *Pseudomonas aeruginosa*, and *Enterobacter* species) and two fungi (*C. albicans* and *C. neoformans)*, with further investigations into the antifungal activities of the Pd complexes against a broader range of isolates including the clinical relevant *Candida auris*.

## Results and Discussion

**Figure 2:**
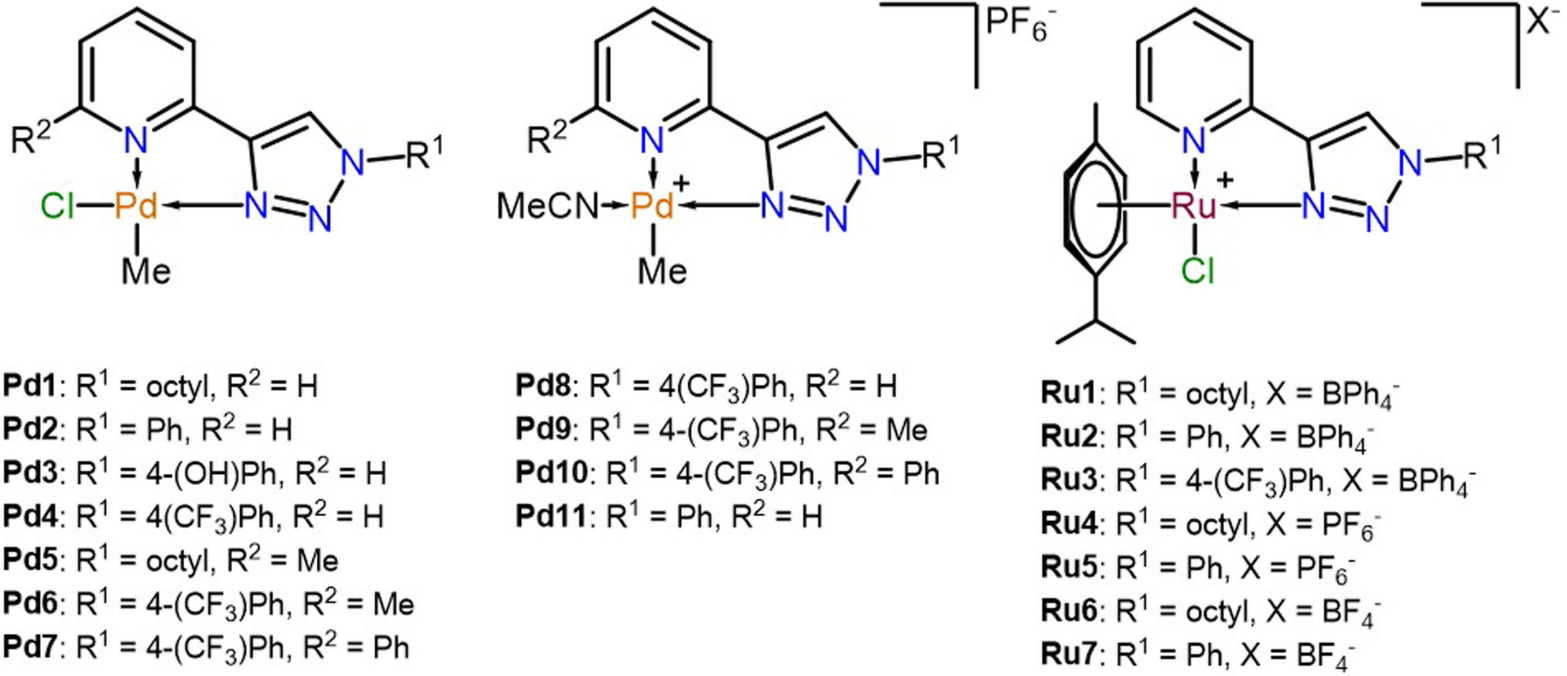
The structures of the complexes evaluated.

The synthesis and characterization of the complexes have been previously reported and are summarized in Figure 2. With help of 2D NMR spectroscopy and single‐crystal X‐ray diffraction (SCXRD), the molecular structure of the Pd complexes was confirmed where the methyl ligand on the metal centre is trans to the pyridine ring.^[16]^ Similarly, SCXRD also confirmed the cationic half‐sandwich structure of the Ru^II^ complexes.^[17]^ Therefore, the synthesis of these complexes will only be discussed briefly.

The 1,2,3‐triazole ligands were prepared through conventional and microwave‐assisted copper‐catalysed alkyne−azide cycloaddition (CuAAC) reactions, better known as “click chemistry”. In the cases where R^2^=Me, the starting alkynyl reagent was prepared in a Sonogashira coupling reaction followed by a deprotection step to obtain 6‐ethynyl−2‐methylpyridine, which was subsequently used in the CuAAC reaction along with the appropriate azide. The phenyl‐substituted ligand, 2‐ethynyl−6‐phenylpyridine, was prepared through a Suzuki‐coupling between 2,6‐dibromopyridine and phenylboronic acid, which was followed by a Sonogashira coupling and the final deprotection step to yield the desired ligands.^[16]^

The neutral 1,2,3‐triazolyl−pyridinato palladium(II)methyl chloride complexes (**Pd1–Pd7**) were obtained by reacting the ligands with the Pd precursor, [(COD]PdMeCl where COD=cyclooctadiene] in DCM to afford the final complexes in moderate to excellent yields (56–89 %). The cationic Pd^II^ complexes, **Pd8–Pd11**, were prepared by reacting the corresponding neutral complex with AgPF_6_ in the presence of acetonitrile to yield the final cationic complexes in excellent yields (81–88 %).^[16]^

The Ru^II^ complexes (**Ru1–Ru7**) were obtained by treating the ligands with [RuCl_2_(*p*‐cymene)]_2_ in DCM for one hour followed by the addition of the desired counterion salt as a solid. The reaction was allowed to stir for an additional 30 min after the salt metathesis, whereafter the product was isolated through trituration with hexane and purified by recrystallization from DCM/hexane. The desired complexes were obtained in excellent yields (80–91 %) and fully characterized as reported previously.^[17]^

### Antimicrobial evaluation

The preliminary antimicrobial screening of the complexes was performed at a fixed concentration of 32 μg mL^−1^ of each complex and the percentage growth relative to the untreated control was measured after 18 h in the presence of the compounds. The initial screening included five bacterial species, *S. aureus* (Sa), *Escherichia coli* (Ec), *K. pneumoniae* (Kp), *P. aeruginosa* (Pa), and *A. baumannii* (Ab), and two fungi, *C. albicans* (Ca) and *C. neoformans* (Cn). Most of the complexes showed some inhibition of the microbial growth rates, as summarized in Table S1 in the Supporting Information, with the exception of **Ru1** and **Ru5** which were therefore not carried over to the minimum inhibition concentration (MIC), cytotoxicity and haemolytic studies.

For the MIC determination, a concentration range of each complex was evaluated against the same microbes with the addition of a human embryonic kidney cell line, HEK‐293 (Hk), to evaluate the toxicity of the complexes, and human red blood cells (RBC) to check for haemolytic properties. The results of these studies are summarized in Table S2. None of the complexes had an MIC below the maximum concentration tested, 32 μg mL^−1^, against either the Gram‐negative or ‐positive bacteria, except for **Pd10**, which showed some activity against MRSA (MIC(**Pd10**)≤0.25 μg mL^−1^). Interestingly, most of the Pd^II^ complexes showed some activity against the fungal strains, while the Ru^II^ complexes bearing similar ligands displayed no significant activity against any of the microbes evaluated. The observation of specifically antifungal activity is promising as it indicates a specific target in fungal pathogens and not general toxicity of the palladium complexes. Notably, none of the complexes were cytotoxic or haemolytic at the highest concentrations tested.

Encouraged by these results, the Pd^II^ complexes were screened against a broader fungal panel including some multidrug‐resistant (MDR) clinical isolates, to determine the extent of the antifungal activity. The extended panel contained four *Candida* and two *Cryptococcus* strains (Table 1). As **Pd3** and **Pd6** displayed poor activity against the fungal strains in the MIC screening test, they were not evaluated against the extended fungal panel. The antifungal MICs [μg mL^−1^] are summarized in Table 1 as a range of MICs from two biological replicates and two technical replicates (*n*=4), with fluconazole as a positive control compound (for MIC in μM, see Table S3).

**Table 1:** The MIC values of the complexes as evaluated against an extended panel of fungi.

	MIC [μg mL^−1^]					
Compounds	*C. albicans*	*C. neoformans*	*Candida tropicalis*	*C. glabrata*	*C. deuterogattii*	*C. auris*
	ATCC 90028	ATCC 208821	ATCC 750	ATCC 90030	ATCC 32609	CBS10913
**Pd1**	0.25–0.5	0.125	0.125	0.06–0.125	0.25	0.03–0.125
**Pd2**	≤0.25	≤0.25	1.0	16	0.016	0.25
**Pd3**	16	0.5	n.d.	n.d.	n.d.	n.d.
**Pd4**	0.5–2	0.125–0.25	0.5–1	1–2	0.25–1	0.06–1
**Pd5**	4–8	0.5–1	8–16	8–16	4–2	4–2
**Pd6**	>32	>32	n.d.	n.d.	n.d.	n.d.
**Pd7**	0.25–1	<0.25	1	0.5–1	0.5–1	0.5–1
**Pd8**	0.5–1	0.06–0.25	0.5	1–2	0.25–0.5	0.25–1
**Pd9**	0.5–1	0.125	0.5–1	0.5–2	0.25–0.5	0.5–2
**Pd10**	0.25–1	0.25	0.5–1	0.5–2	0.5–2	0.5–2
**Pd11**	2–4	1	2–4	8	2	1–4
fluconazole	0.25–0.5	8	4	>32	>32	≥32

All the complexes showed activity to varying degrees against all the strains tested, and more importantly, many of the compounds had MICs below that of fluconazole against *C. neoformans*, *Candida glabrata*, *Cryptococcus deuterogattii* and *C. auris*. In contrast, the free ligands tested (Table S4) generally showed no activity against the same strains, suggesting that any observed activity is indeed due to the metal complex and not a ligand that is released.

**Pd1**, **Pd4**, and **Pd7–10** showed good broad spectrum antifungal activity against all the fungal strains tested with the highest MIC obtained against *C. albicans*. Interestingly, **Pd5** which is analogous to **Pd1** with the addition of a methyl in the R^2^ position, was not as potent as **Pd1**; this suggests that the methyl group on the pyridyl might have a more pronounced influence than anticipated. In the cases where the triazole substituent R^1^ is changed to a phenyl ring (**Pd2** as a neutral complex, and **Pd11** as a cationic complex) and R^2^ remains an H‐atom, the cationic **Pd11** is revealed as less potent than the neutral complex, **Pd2**. It should also be noted that **Pd2** was the most selective complex towards *C. deuterogattii* with MIC=0.016 μg mL^−1^.

The activity of the 4‐(CF_3_)Ph‐bearing complexes can be divided into the cationic versus neutral complexes and the influence of the R^2^ substituents. Where R^2^=H, the activity profiles of **Pd4** and **Pd8** fall within the same ranges against each of the fungal strains tested, which might suggest that the cationic nature of **Pd8** does not have a significant influence on the activity of the complex, which is contrary to the trend seen between **Pd2** and **Pd11**. This suggests that the strong electron withdrawing properties of the *para*‐CF_3_ substituent on the triazole bound phenyl ring has a significant influence on the activity of the compounds. When compared to the R^1^=Ph complexes (**Pd2** and **Pd11**), the inclusion of the electron‐withdrawing group (EWG) results in a lower MIC and negates the influence of the charge of the overall complex on the activity of the compounds. This argument is supported by the fact that **Pd3**, which included an electron‐donating −OH on the triazole bound phenyl ring, was significantly less active than the more electron‐withdrawing substituents and therefore not evaluated against the extended fungal panel.

Next, when comparing the 4‐(CF_3_)Ph‐substituted compounds based on their R^2^ substituents, we see that the inclusion of a methyl group on the pyridine ring resulted in an inactive neutral complex, **Pd6**, which was not evaluated against the extended fungal panel. However, when R^2^=Ph, **Pd7**, good antifungal activity is recovered. Therefore, where the inclusion of the methyl group is concerned, it appears that it causes a decrease in the activity of the neutral complexes, as seen in **Pd1** versus **Pd5** and **Pd4** versus **Pd6** but has no influence on the activity of the cationic complexes. While when R^2^=Ph that activity is restored, which suggests that a bulky EWG in the R^2^ position favors the antifungal activity of the complexes.

When comparing cationic 4‐(CF_3_)Ph‐substituted complexes based on their R^2^ substituents, the MIC values have similar activity profiles against all the fungal strains tested with no clear dependence on the R^2^ groups. Nonetheless, all the cationic 4‐(CF_3_)Ph‐substituted complexes showed improved activities against the fungal strains tested relative to the fluconazole control, with **Pd8** showing MIC=0.06–0.26 μg mL^−1^ against *C. neoformans*, which was the most potent complex evaluated against that strain.

### Solubility and stability

For biological applications, it is necessary to investigate the stability and solubility of the compounds in DMSO and aqueous media. During the initial investigation of the compounds, it was determined that only complexes **Pd1** and **Pd5** with the octyl substituents were soluble in chlorinated solvents and DMSO, while the rest of the neutral complexes were only soluble in DMSO. The cationic complexes were fully soluble in DMSO and acetonitrile, and partially soluble in chlorinated solvents.^[16]^

To assess the solubility of the complexes in the presence of an aqueous buffer, turbidimetric assays were performed to determine the kinetic solubility ranges of each complex in a 2 % DMSO/PBS solution.[^25^, ^26^] The results of the turbidimetric assays are summarized in Table 2.

**Table 2:** The kinetic solubility ranges of the compounds at 25 °C as determined in a turbidimetric assay.

Compound	Solubility range in 100 % DMSO		Solubility range in 2 % DMSO/PBS	
	μM	μg mL^−1^	μM	μg mL^−1^
**Pd1**	>200	>83.0	5–10	2.18–4.20
**Pd2**	>200	>75.8	20–40	7.58–15.2
**Pd3**	>200	>79.0	20–40	7.90–15.8
**Pd4**	>200	>89.4	20–40	8.94–17.9
**Pd5**	>200	>85.9	5–10	2.15–4.29
**Pd7**	>200	>104.7	10–20	5.23–10.47
**Pd8**	>200	>119.5	10–20	5.98–11.95
**Pd9**	>200	>122.4	80–160	48.9–97.9
**Pd10**	>200	>134.8	10–20	6.74–13.5
**Pd11**	>200	>105.9	20–40	10.6–21.2

The stability of the complexes in the presence of DMSO was also confirmed using complex **Pd4**. The UV‐vis spectrum of **4** dissolved in excess DMSO was monitored over time at 25 °C and no change in the spectrum was observed over a 24‐hour period (Figure S1). From this study we can conclude that this complex is stable in the presence of DMSO. It should be noted that the stability of the other complexes might differ slightly. However, as the free ligands did not show any antifungal activity (Table S4) it can be inferred that the compounds are stable enough to elicit the antifungal effect observed in the measurement time‐frame.

Interestingly, the least soluble complex, **Pd1**, showed the best broad‐spectrum activity of all the compounds evaluated, which emphasizes the importance of lipophilicity in drug development. However, no direct correlations are seen between the solubility of the compounds relative to the activity of the compounds.

### In vivo studies

Having established that Pd^II^−pyta complexes display broad‐spectrum antifungal activity with no in vitro toxicity, we moved on to assessing the in vivo toxicity of representative metal complexes in the greater wax moth *Galleria mellonella* (complexes **Pd1–3** could not be included in these studies as not enough material was left over). The larvae of this insect has been previously used for antimicrobial toxicity studies and infection studies and provides a low‐cost, high‐throughput animal model with data correlating well with that obtained from the more ethically controversial rodent models.[^27^, ^28^]

Three groups of 5 *G. mellonella* larvae (250–270 mg) were injected with 10 μL of highest solubility concentration (Table S4) and incubated at 37 °C, to text the toxicity of each test compound, in either 2 or 10 % DMSO, with a control group injected with either 2 or 10 % DMSO alone. The survival of the larvae was followed for 6 days after the injection and all compounds were established as “nontoxic”, as 100 % of larvae survived over the monitoring period. A table containing the results from the in vivo toxicity studies is contained in the supplementary information (Table S4).

## Conclusions

A series of Pd^II^− and Ru^II^−pyridyl−1,2,3‐triazole complexes were successfully screened against the ESKAPE pathogens in collaboration with CO‐ADD. While the Ru^II^ complexes proved to be inactive, the Pd^II^ complexes showed activity against the fungal strains in the initial MIC‐determination steps. These complexes were then evaluated against an extended panel of fungi and showed broad‐spectrum activity. Most promising is **Pd1** (R^1^=octyl, R^2^=H), which was not only a vast improvement on the control compound, fluconazole, but also displayed potent activity against *C. glabrata*, which is considered to be the most antifungal resistant strain in the extended fungal panel.

Additionally, the toxicity of the compounds was assessed in vitro against HEK‐293 cells and in vivo in *G. mellonella* larvae, where no toxicity was observed, even at higher drug concentrations.

This work demonstrates the versatility of pyta ligands and visualizes the impact each substituent on the ligand has on the antimicrobial activity of the compounds. Furthermore, it opens up the world of investigating Pd^II^ complexes as antifungal agents by showing the low toxicity of the compounds accompanied by broad‐spectrum activity that surpasses that of the current clinically applied treatments. Future work will aim to synthesize new derivatives with improved solubility with the goal of optimizing the treatment of fungal infection in vivo.

## Experimental Section

**Materials and methods**: The synthesis and characterization of complexes **Pd1–Pd11** ^[16]^ and **Ru1–Ru7** ^[17]^ were reported previously since they were initially developed as transition metal catalysts. Compounds were prepared at 10 mg mL^−1^ in 100 % DMSO and stored at 4 °C until used. The compounds were manually serially diluted in 20 % DMSO two‐fold down the wells of a Mother Plate 384‐deep well PP plate. Then, 5 μL per well was stamped into Test Plates (384‐well NBS plates) in duplicate per assay.

**Antibacterial data collection**: Inhibition of bacterial growth was determined measuring absorbance at 600 nm (OD_600_), using a Tecan M1000 Pro monochromator plate reader. The percentage of growth inhibition was calculated for each well, using the negative control (medium only) and positive control (bacteria without inhibitors) on the same plate as references.

For all the bacterial assays, each bacterial strain was cultured in Cation‐adjusted Mueller Hinton broth (CAMHB; Bacto Laboratories 212322) at 37 °C overnight. A sample of each culture was then diluted 40‐fold in fresh CAMHB and incubated at 37 °C for 1.5–3 h. The resultant mid‐log phase cultures were diluted with CAMHB (CFU mL^−1^ measured by OD_600_), then added to each well of the compound‐containing plates (384‐well non‐binding surface (NBS) plates; Corning CLS3640), giving a cell density of 5×10^5^ CFU mL^−1^ and a total volume of 50 μL (<2 % DMSO). Plates were covered and incubated at 37 °C for 18 h without shaking. Inhibition of bacterial growth was determined measuring absorbance at 600 nm (OD_600_), using media only as negative control and bacteria without inhibitors as positive control. MIC values were determined as the lowest concentration at which the growth was inhibited at≥80 % (equivalent to no visible growth by eye). Colistin sulfate (Sigma C4461) and vancomycin HCl (Sigma 861987) were used as internal controls on each plate for Gram‐negative and Gram‐positive bacteria, respectively. All compounds were tested as 2 independent biological replicates with 2 technical repeats each.

Percentage growth inhibition of an individual sample is calculated based on Negative controls (media only) and Positive Controls (bacterial/fungal media without inhibitors). Please note negative inhibition values indicate that the growth rate (or OD_600_) is higher compared to the negative control (bacteria/fungi only, set to 0 % inhibition). The growth rates for all bacteria and fungi has a variation of ±10 %, which is within the reported normal distribution of bacterial/fungal growth. Any significant variation (or outliers/hits) is identified by the modified *Z*‐score, and actives are selected by a combination of inhibition value and *Z*‐score analysis. *Z*‐score analysis is done to investigate outliers or hits among the samples. The *Z*‐score is calculated based on the sample population using a modified *Z*‐score method which accounts for possible skewed sample population.

**Antifungal data collection**: Fungi strains were cultured for 3 days on yeast extract‐peptone dextrose (YPD) agar at 30 °C. A yeast suspension of 1×10^6^ to 5×10^6^ CFU mL^−1^ (as determined by OD_530_) was prepared from five colonies. The suspension was diluted in supplemented YNB and added to each well of the compound‐containing plates giving a final cell density of fungi suspension of 2.5×10^3^ CFU mL^−1^ and 2 % DMSO. All plates were covered and incubated at 35 °C for 36 h without shaking.

Two biological replicates x2 technical replicates were conducted on separate days (final *n*=4).

Growth inhibition of *C. albicans, C. tropicalis and C. glabrata* was determined by measuring absorbance at 630 nm (OD_630_), while the growth inhibition of *Cryptococcus* spp. and *C. auris* was determined by measuring the difference in absorbance between 600 and 570 nm (OD_600‐570_), after the addition of resazurin (0.01 % final concentration) and incubation at 35 °C for 2 h. The absorbance was measured using a Biotek Multiflo Synergy HTX plate reader.

The percentage of growth inhibition was calculated for each well, using the negative control (media only) and positive control (fungi without inhibitors) on the same plate. The MIC was determined as the lowest concentration at which the growth was fully inhibited, defined by an inhibition ≥80 %. In addition, the maximal percentage of growth inhibition is reported as *D*_Max_, indicating any compounds with marginal activity.

**Cytotoxicity data collection**: HEK‐293 ATCC CRL‐1573 human embryonic kidney cells were counted manually in a Neubauer haemocytometer and added to compound‐containing plates (384‐well plates, tissue culture treated (TC); Corning CLS3712) giving a final density of 5000 cells/well in a and a total volume of 50 μL (<0.5 % DMSO), using Dulbecco's modified Eagle's medium (DMEM; Life Technologies 11995–073) with 10 % foetal bovine serum (FBS; GE SH30084.03). The cells were incubated together with the compounds for 20 h at 37 °C in 5 % CO_2_. Cytotoxicity (or cell viability) was measured by fluorescence, *λ*_ex_=560/10 nm, *λ*_em_=590/10 nm (F560/590), after addition of 5 μL of 25 μg mL^−1^ resazurin (2.3 μg mL^−1^ final concentration; Sigma R7017) and after further incubation for 3 h at 37 °C in 5 % CO_2_, using media only as negative control and cells without inhibitors as positive control. CC_50_ (concentration at 50 % cytotoxicity) were calculated by curve fitting the inhibition values vs. log(concentration) using a sigmoidal dose‐response function, with variable fitting values for bottom, top and slope. Tamoxifen (Sigma T5648) was used as internal control on each plate.

Growth inhibition of HEK293 cells was determined measuring fluorescence at *λ*_ex_=530/10 nm and *λ*_em_=590/10 nm (F560/590), after the addition of resazurin (25 μg mL^−1^ final concentration) and incubation at 37 °C and 5 % CO_2_, for an additional 3 h. The fluorescence was measured using a Tecan M1000 Pro monochromator plate reader. The percentage of growth inhibition was calculated for each well, using the negative control (medium only) and positive control (cell culture without inhibitors) on the same plate as references. Concentration at 50 % cytotoxicity (CC_50_) values were calculated by fitting the curve of the inhibition values vs. log(concentration) using Sigmoidal dose‐response function, with variable values for bottom, top and slope. The curve fitting is implemented using Pipeline Pilot's dose‐response component (giving similar results to similar tools such as GraphPad′s Prism and IDBS′s XlFit). Any value with > indicates a sample with no activity (low *D*_Max_ value) or samples with CC_50_ values above the maximum tested concentration (higher *D*_Max_ value)

**Haemolysis data collection**: Human whole blood (Australian Red Cross) was washed three times with 3 volumes of 0.9 % NaCl and resuspended in a concentration of 0.5×10^8^ cells mL^−1^, determined by manual cell count in a Neubauer haemocytometer. Washed cells were added to compound‐containing plates (384‐well polypropylene plates (PP); Corning 3657) for a final volume of 50 μL, shaken and incubated for 1 h at 37 °C. After incubation, the plates were centrifuged at 1000 *g* for 10 min to pellet cells and debris, 25 μL of the supernatant was then transferred to reading plates (384‐well, polystyrene plated (PS), Corning CLS3680), with haemolysis determined by measuring the supernatant absorbance at 405 nm (OD_405_), using cells without inhibitors as negative control and cells with 1 % Triton X‐100 (Sigma T8787) as positive control. HC_10_ and HC_50_ (concentration at 10 % and 50 % haemolysis, respectively) were calculated by curve fitting the inhibition values vs. log(concentration) using a sigmoidal dose‐response function with variable fitting values for top, bottom and slope. Melittin (Sigma M2272) was used as internal control on each plate. The use of human blood (sourced from the Australian Red Cross Blood Service) for haemolysis assays was approved by the University of Queensland Institutional Human Research Ethics Committee, Approval Number 2014000031.

Concentration at 10 % haemolytic activity (HC_10_) values were calculated by fitting the curve of the inhibition values vs. log(concentration) using Sigmoidal dose‐response function, with variable values for bottom, top and slope. The curve fitting is implemented using Pipeline Pilot's dose‐response component (giving similar results to similar tools such as GraphPad′s Prism and IDBS′s XlFit). The curve fitting resulted in HC_50_ (50 %) values, which are converted into HC_10_ by HC_10_=HC_50_×(10/90) (1/slope); Any value with > indicates a sample with no activity (low *D*_Max_ value) or samples with HC_10_ values above the maximum tested concentration (higher *D*_Max_ value).

***G. mellonella*** **in vivo toxicity assay**: The toxicity of compounds was tested in vivo using the *G. mellonella* model using our previously described methods.^[12]^ Briefly, *G. mellonella* larvae were reared in Controlled Environmental Chambers (Steridium) at Macquarie University (Sydney, Australia) at 30 °C and 65 % humidity with a 12‐h light/dark cycle. Larvae (250–270 mg) were individually injected with 10 μL of chemical compound into the last right proleg using a 100 μL syringe (Hamilton Ltd). Each compound was dissolved in 2 % DMSO at the maximum concentration of 100 μM and diluted in 10 % DMSO to increase the solubility of the compounds. We injected 5 larvae in triplicate for all the compounds at the highest testing concentrations (Table S4). Larvae injected with 2 % and 10 % DMSO were included as negative controls. Following injection, the larvae were incubated at 37 °C for 6 days and monitored every 24 h for health and survival according to the *G. mellonella* Health Index Scoring System.[^27^, ^28^, ^29^]

Deposition Numbers 1921497 (for **Pd1**), 1921498 (for **Pd3**), 1921499 (for **Pd4**), 1921500 (for **Pd6**), and 1969487 (for **Pd10**) contain the supplementary crystallographic data for this paper. These data are provided free of charge by the joint Cambridge Crystallographic Data Centre and Fachinformationszentrum Karlsruhe Access Structures service.

## Conflict of interest

The authors declare no conflict of interest.

1

## Biographical Information

*Angelo Frei did his master's degree on ruthenium polypyridyl complexes as photosensitizers at the University of Zurich with Prof. Gilles Gasser. He got his PhD in 2018 with Prof. Roger Alberto on the development of multifunctional cyclopentadiene ligands for theranostic applications. He joined Prof. Mark Blaskovich's group at the University of Queensland on a Swiss National Science Foundation Early.Postdoc Mobility Fellowship. There he investigated metal complexes as potential antimicrobial agents. In 2020, he joined Prof. Nicholas Long's group at Imperial College London to work on radioimaging agents, before being awarded a SNSF Ambizione Fellowship at the University of Bern in 2022 where he is a Junior Group Leader. His research involves exploring metal‐containing compounds as antimicrobial agents and their mechanism of action*.

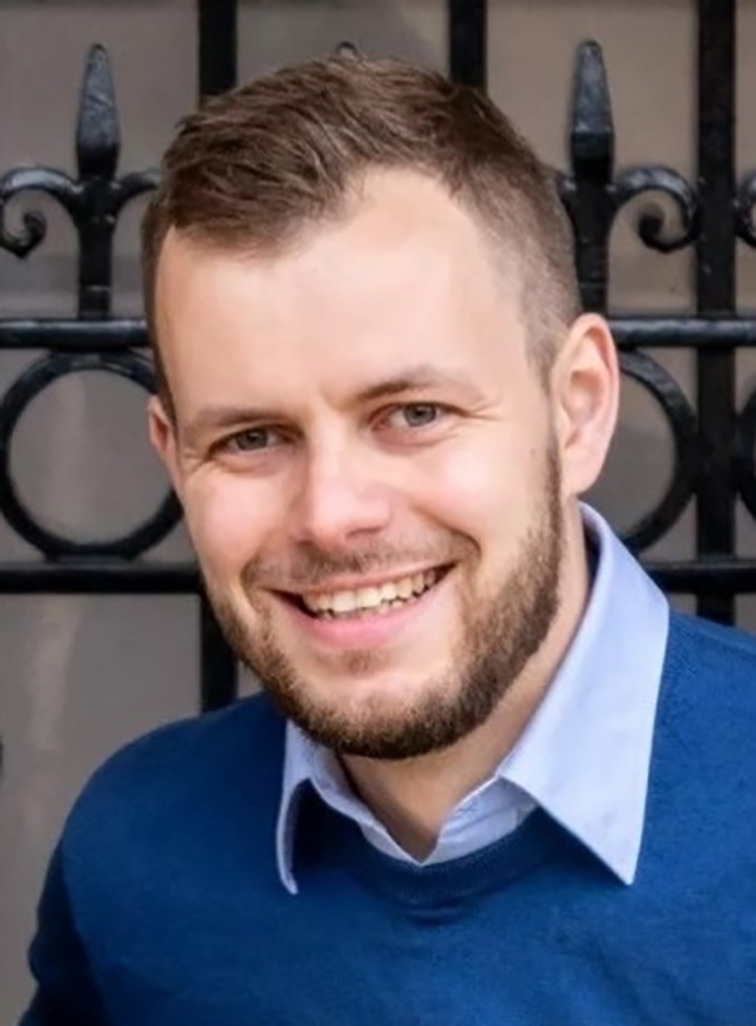

## Supporting information

As a service to our authors and readers, this journal provides supporting information supplied by the authors. Such materials are peer reviewed and may be re‐organized for online delivery, but are not copy‐edited or typeset. Technical support issues arising from supporting information (other than missing files) should be addressed to the authors.

Supporting Information

## Data Availability

The data that support the findings of this study are available in the supplementary material of this article.
