# Supplementary material for: The Antimicrobial Properties of PdII− and RuII−pyta Complexes
Source: Chembiochem. 2023 Sep 5;24(19):e202300247. doi: 10.1002/cbic.202300247 (PMC10947176; doi:10.1002/cbic.202300247)
Supplement: Supplementary file 1 — Supporting Information [file CBIC-24-0-s001.pdf]

ChemBioChem

Supporting Information

The Antimicrobial Properties of Pd^{II}– and Ru^{II}–pyta Complexes

Annick van Niekerk,* M. Cassiem Joseph, Angela Kavanagh, Hue Dinh, Andrew J. Swarts, Selwyn F. Mapolie, Johannes Zuegg, Amy K. Cain, Alysha G. Elliott, Mark A. T. Blaskovich, and Angelo Frei*

Preliminary antimicrobial screening against the ESKAPE pathogens.

Table S1: The results from the preliminary screening of the pyta complexes at a single concentration against a selection of bacteria and fungi.

Compound name	Percentage growth inhibition*						
	Bacteria (Gram+)	Bacteria (Gram-)				Fungi	
	Staphylococcus aureus (Sa) ATCC 4330	*Escherichia coli* (Ec) ATCC 25922	*Klebsiella pneumoniae* (Kp) ATCC 700603	*Pseudomonas aeruginosa* (Pa) ATCC 27853	*Acinetobacter baumannii* (Ab) ATCC 19606	*Candida albicans* (Ca) ATCC 90028	*Cryptococcus neoformans* (Cn) ATCC 208821
**Pd1**	97.99	18.2	18.38	-6.42	63.33	105.36	114.35
**Pd2**	3.61	29.08	20.37	15.76	58.21	95.63	105.2
**Pd3**	-26.8	25.43	22.15	15.24	41.22	108.47	120.58
**Pd4**	66.52	40.95	17.85	11.01	32.96	101.07	100
**Pd5**	53.27	43.8	19.15	13.14	53.7	104.4	109.56
**Pd6**	91.29	0.13	1.36	8.12	30.87	4.01	7.66
**Pd7**	-6.64	18.88	13.04	18.83	35.6	95.75	135.76
**Pd8**	-44.67	14.06	17.03	15.66	26.14	99.74	106.65
**Pd9**	-0.14	11.53	11.6	10.09	34.41	94.84	103.95
**Pd10**	11.66	31.24	29.94	73.72	42.63	96.63	127.08
**Pd11**	-7.55	31.76	16.11	15.72	49.84	97.45	105.65
**Ru1**	-9.41	-1.21	6.15	7.15	5.76	62.11	-36.56
**Ru2**	45.94	17.72	10.36	1.96	42.93	99.71	-40.12
**Ru3**	-20.72	-1.06	4.1	8.91	6.35	82.95	-20.48
**Ru4**	98.27	34.53	18.32	14.42	44.63	5.99	106.99
**Ru5**	-19.55	6.55	6.67	11.3	24.68	4	-17.88
**Ru6**	97.04	12.16	13.38	14.77	35.28	6.66	102.49
**Ru7**	-16.84	11.67	12.42	18.83	35.24	5.33	-26.6

* Initial screening was performed at compound concentrations of 32.00 µg/mL. The percentage growth rate was calculated relative to the negative control (media only), therefore negative values indicate bacterial growth in the presence of the compounds.

MIC determination, cytotoxicity, and Haemolysis determination

Table S2: The MIC values of the complexes evaluated against the ESKAPE pathogens as well as the human embryonic kidney cells (HEK-293) and human red blood cells (RBC) to assess the cytotoxicity and haemolytic properties of the compounds.

	Minimum inhibition concentration (µg/mL)								
	Bacteria (Gram+)	Bacteria (Gram-)				Fungi		HEK-293	RBC
	Sa	Ec	Kp	Pa	Ab	Ca	Cn	Hk	Hm
**Pd1**	32	>32	>32	>32	>32	2	0.5	32	>32
**Pd2**	>32	>32	>32	>32	>32	<=0.25	<=0.25	>32	>32
**Pd3**	>32	>32	>32	>32	>32	16	0.5	>32	>32
**Pd4**	>32	>32	>32	>32	>32	2	<=0.25	>32	>32
**Pd5**	>32	>32	>32	>32	>32	<=0.25	1	>32	>32
**Pd6**	>32	>32	>32	>32	>32	>32	>32	>32	>32
**Pd7**	>32	>32	>32	>32	>32	<=0.25	<=0.25	>32	>32
**Pd8**	>32	>32	>32	>32	>32	<=0.25	<=0.25	>32	>32

**Pd9**	>32	>32	>32	>32	>32	<=0.25	<=0.25	>32	>32
**Pd10**	<=0.25	>32	>32	>32	>32	<=0.25	<=0.25	>32	>32
**Pd11**	>32	>32	>32	>32	>32	<=0.25	<=0.25	>32	>32
**Ru2**	>32	>32	>32	>32	>32	>32	>32	>32	>32
**Ru3**	>32	>32	>32	>32	>32	>32	>32	>32	>32
**Ru4**	32	>32	>32	>32	>32	>32	8	32	>32
**Ru6**	>32	>32	>32	>32	>32	>32	>32	>32	>32
**Ru7**	>32	>32	>32	>32	>32	>32	>32	>32	>32

MIC (µM) against the extended fungal panel

Table S3: The MIC (µM) concentrations of the compounds evaluated against the extended fungal panel.

**Compound name**	**MIC (µM)**					
	Candida albicans **ATCC 90028**	***Cryptococcus neoformans*** **ATCC 208821**	***Candida tropicalis*** **ATCC 750**	***Candida glabrata*** **ATCC 90030**	***Cryptococcus deuterogattii*** **ATCC 32609**	***Candida auris*** **CBS10913**
**Pd1**	0.602 - 1.20	0.301	0.301	0.144 - 0.301	0.602	0.0722 - 0.301
**Pd2**	<0.659	<0.659	2.64	42.2	0.0422	0.659
**Pd3**	40.5	1.27	n.d.	n.d.	n.d.	n.d.
**Pd4**	1.12 - 4.47	0.280 - 0.559	1.12 - 2.24	2.24 - 4.47	0.559 - 2.24	0.13 - 2.24
**Pd5**	9.32 - 18.6	1.16 - 2.33	18.6 - 37.3	18.6 - 37.3	4.66 - 9.32	4.66 - 9.32
**Pd6**	>69.4	>69.4	n.d.	n.d.	n.d.	n.d.
**Pd7**	0.478 - 1.91	<0.478	1.91	0.956 - 1.91	0.956 - 1.91	0.956 - 1.91
**Pd8**	0.837 - 1.67	0.100 - 0.435	0.837	1.67 - 3.35	0.418 - 0.837	0.418 - 1.67
**Pd9**	0.817 - 1.63	0.204	0.817 - 1.63	0.817 - 3.27	0.409 - 0.817	0.817 - 3.27
**Pd10**	0.371 - 1.48	0.371	0.742 - 1.48	0.742 - 2.97	0.742 - 2.97	0.742 - 2.97
**Pd11**	3.78 - 7.55	1.89	3.78 - 7.55	15.1	3.78	1.89 - 7.55
fluconazole	0.816 - 1.63	26.1	13.1	>104	>104	>104

In vivo toxicity evaluation

Table S4: The results from the in vivo toxicity studies in G. mellonella larvae.

**Compound name**	**Concentration in 2% DMSO**	**Toxicity result (1)**	**Concentration in 10% DMSO**	**Toxicity test result (2)**
**Pd4**	40 µM	Non-toxic	100 µM	Non-toxic
**Pd5**	10 µM	Non-toxic	100 µM	Non-toxic
**Pd6**	160 µM	Non-toxic	1000 µM	Non-toxic
**Pd7**	20 µM	Non-toxic	100 µM	Non-toxic
**Pd8**	20 µM	Non-toxic	100 µM	Non-toxic
**Pd9**	160 µM	Non-toxic	1000 µM	Non-toxic
**Pd10**[a]	NA	NA	NA	NA

**Pd11**	40 µM	Non-toxic	100 µM	Non-toxic
DMSO	2%	Non-toxic	10%	Non-toxic

[a] The complex did not fully dissolve at the concentrations tested and was therefore not injected into the larvae.

Stability of Pd4 in DMSO

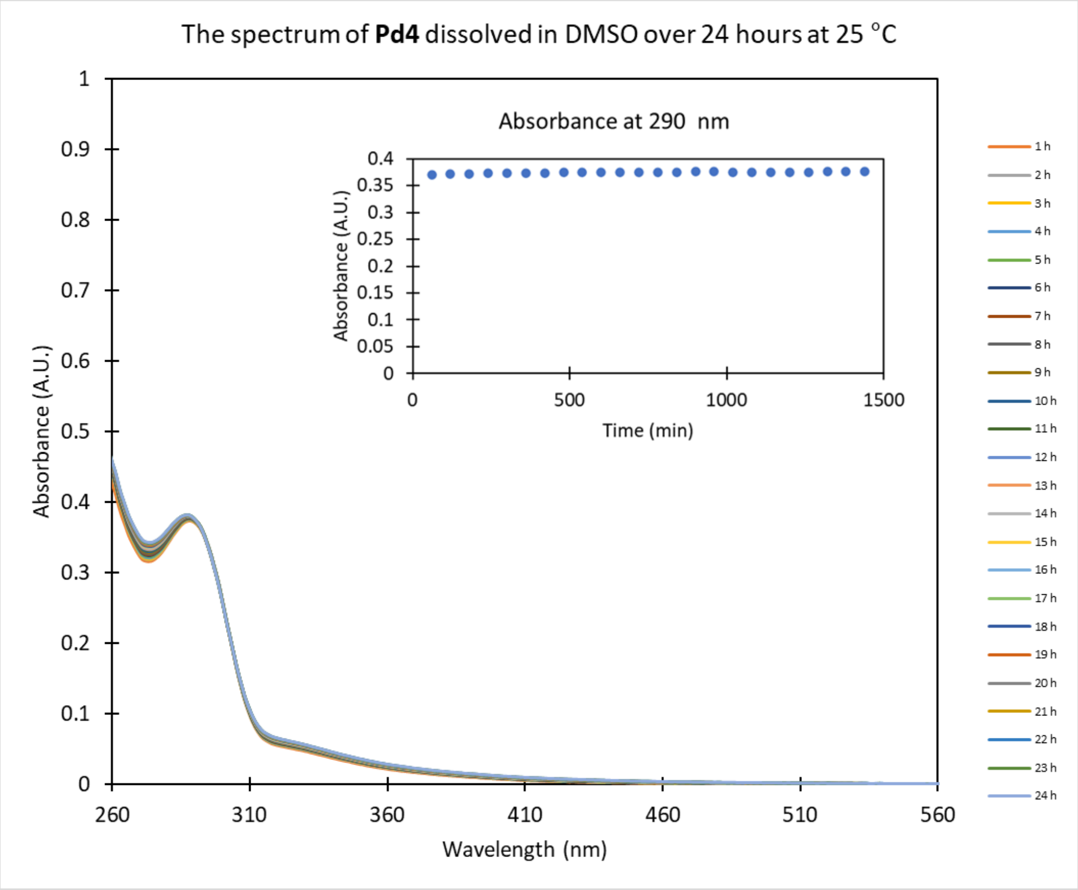

Figure S1: The stability of Pd4 in DMSO measured over time using the UV-vis spectroscopy.

Table S4: The MIC (µM) concentrations of the ligands and starting palladium compound evaluated against the extended fungal panel.

**Compound name**	**MIC (µM)**					
	Candida albicans **ATCC 90028**	***Cryptococcus neoformans*** **ATCC 208821**	***Candida tropicalis*** **ATCC 750**	***Candida glabrata*** **ATCC 90030**	***Cryptococcus deuterogattii*** **ATCC 32609**	***Candida auris*** **CBS10913**
**L1**	>32.0	>32.0	>32.0	>32.0	>32.0	>32.0
**L2**	>32.0	>32.0	>32.0	>32.0	>32.0	2.00
**L4**	>16.0	>16.0	>16.0	>16.0	>16.0	>16.0
**L5**	>32.0	>32.0	>32.0	>32.0	>32.0	>32.0
**L6**	>32.0	>32.0	>32.0	>32.0	>32.0	>32.0
**L7**	>32.0	>32.0	>32.0	>32.0	>32.0	>32.0

**L8**	>32.0	>32.0	>32.0	>32.0	>32.0	>32.0
fluconazole	0.816 - 1.63	26.1	13.1	>104	>104	>104

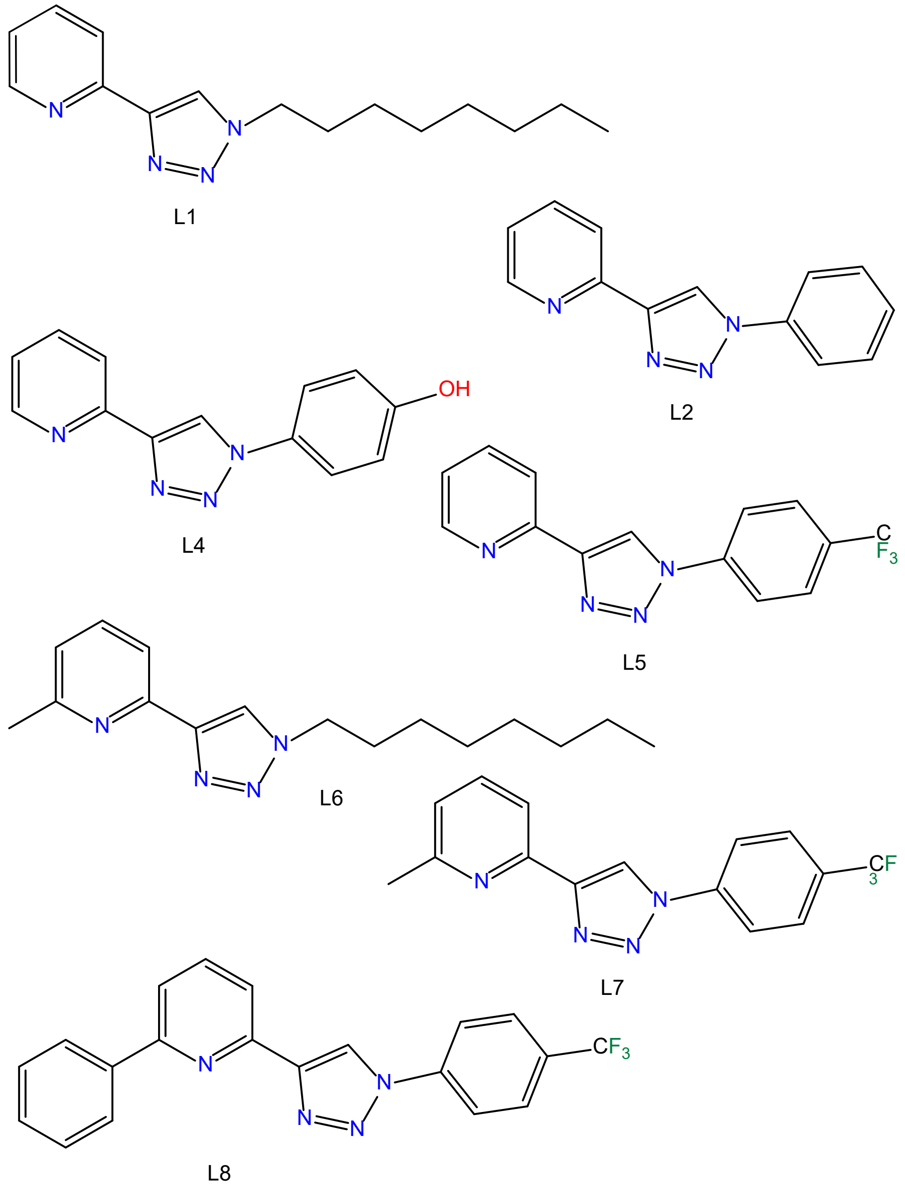

Figure S1: Structures of the tested ligands. (Ligand L3 was not available anymore)
